# Serotonin is a Common Thread Linking Different Classes of Antidepressants

**DOI:** 10.21203/rs.3.rs-2741902/v1

**Published:** 2023-03-28

**Authors:** Colby E. Witt, Sergio Mena, Jordan Holmes, Melinda Hersey, Anna Marie Buchanan, Brenna Parke, Rachel Saylor, Lauren E. Honan, Shane N. Berger, Sara Lumbreras, Frederik H. Nijhout, Michael C. Reed, Janet Best, James Fadel, Patrick Schloss, Thorsten Lau, Parastoo Hashemi

**Affiliations:** 1 Department of Chemistry and Biochemistry, University of South Carolina, Columbia, SC, USA; 2 Department of Bioengineering, Imperial College London, London, United Kingdom; 3 Department of Pharmacology, Physiology and Neuroscience, University of South Carolina School of Medicine, Columbia, SC, USA; 4 Department of Biology, Duke University, Durham, NC, USA; 5 Department of Mathematics, Duke University, Durham, NC, USA; 6 Department of Mathematics, The Ohio State University, Columbus, OH, USA; 7 Department of Psychiatry and Psychotherapy, Biochemical Laboratory, Central Institute of Mental Health, Medical Faculty, Mannheim, Heidelberg University, Mannheim, Germany; 8 Department of Neuroanatomy, Mannheim Centre for Translational Neuroscience, Medical Faculty Mannheim, Heidelberg University, Mannheim, Germany

**Keywords:** depression, SSRIs, NRIs, ketamine, voltammetry, neurogenesis, monoamine hypothesis

## Abstract

Depression pathology remains elusive. The monoamine hypothesis has placed much focus on serotonin, but due to the variable clinical efficacy of monoamine reuptake inhibitors, the community is looking for alternative therapies such as ketamine (synaptic plasticity and neurogenesis theory of antidepressant action). There is evidence that different classes of antidepressants may affect serotonin levels; a notion we test here. We measure hippocampal serotonin in mice with voltammetry and study the effects of acute challenges of antidepressants. We find that pseudo-equivalent doses of these drugs similarly raise ambient serotonin levels, despite their differing pharmacodynamics because of differences in Uptake 1 and 2, rapid SERT trafficking and modulation of serotonin by histamine. These antidepressants have different pharmacodynamics but have strikingly similar effects on extracellular serotonin. Our findings suggest that serotonin is a common thread that links clinically effective antidepressants, synergizing different theories of depression (synaptic plasticity, neurogenesis and the monoamine hypothesis).

## Introduction

Depression is one of society’s most critical health issues. Antidepressants are some of the most prescribed medicines globally, differ significantly in their structure and functions, carry with them side effects and a clinical regime where antidepressant type and dosage are altered on a trial-and-error basis(1). Importantly, there is quite some speculation about whether clinical responses to antidepressants are significantly meaningful with respect to placebo, exercise or psychotherapy(2, 3). The community has not settled on a unifying pathophysiological basis of depression, as such it is nearly impossible to systematically develop novel, more effective antidepressants. To improve a drug’s efficacy, it is critical to identify a measurable biomarker of disease and to investigate the response of the biomarker to potential therapeutics. For depression, much traditional focus was on serotonin, spurred by the monoamine hypothesis of depression(1). Over the last 3 decades, the serotonin hypothesis has moved in and out of spotlight, recently enjoying a resurgence because of clinical trials targeting depression with psychedelics (with affinity for serotonin receptors)(4). In recent years the community has looked for alternative theories of depression and treatment strategies, including other monoamines (*e.g.*, norepinephrine) and the promotion of synaptic plasticity and neurogenesis *via* ketamine’s effects on NMDA receptors(5). Despite the surface-level differences in the mode of action of different classes of antidepressants (including norepinephrine reuptake inhibitors (NRIs) and ketamine), there is emerging evidence that these agents may indirectly target serotonin(6–9).

In this work, we used *in vivo* voltammetry to measure hippocampal serotonin in mice (fast scan cyclic voltammetry (FSCV) and fast scan controlled-adsorption voltammetry (FSCAV)) and studied the effects of acute challenge with two different SSRIs (fluoxetine and escitalopram), an NRI (reboxetine) and ketamine. We found that pseudo-equivalent doses of these drugs raised ambient serotonin levels with similar magnitude and kinetics, despite their very different pharmacodynamics. We then took in-depth experimental and analysis approaches to understand how these drugs modulate the serotonin system. We observed innate, low frequency (several minutes) oscillations in the concentration of ambient serotonin that changed after SSRIs and the NRI, but not after ketamine. We hypothesized that changes were due to differences in Uptake 1 and Uptake 2 mediation of serotonin reuptake. We verified this hypothesis *via* Michaelis-Menten (M-M) analysis of experimental data from evoked release of serotonin with FSCV and showed that inhibition of SERTs (Uptake 1) is intrinsic to SSRIs and that inhibition of Uptake 2 transporters follows the NRI. Furthermore, we found critical differences between fluoxetine and escitalopram. The response to escitalopram was dynamic and did not reach steady state, an effect we hypothesized to be due to rapid shuttling of SERTs in and out of cell membranes. Proof of principle for this notion was provided in a cellular model of serotonin transmission in serotonergic neurons. Finally, we suggested that the effect of ketamine on serotonin may be *via* inverse histaminergic modulation of serotonin and showed *via* further experiments that ketamine is a potent inhibitor of evoked histamine.

Although these drugs, which are all considered antidepressants, have very different pharmacodynamics, they have in common direct or indirect effects on serotonin. This finding signifies serotonin may synergize the different theories of depression and that the community should consider the heterogeneous nature of how depression affects serotonin and its analogues when looking at future design of drugs.

## Results

Full values and statistics can be found in the extended data section in the SI.

### How Do Antidepressants with Different Modes of Action Affect Ambient Extracellular Serotonin?

**Figure 1.**
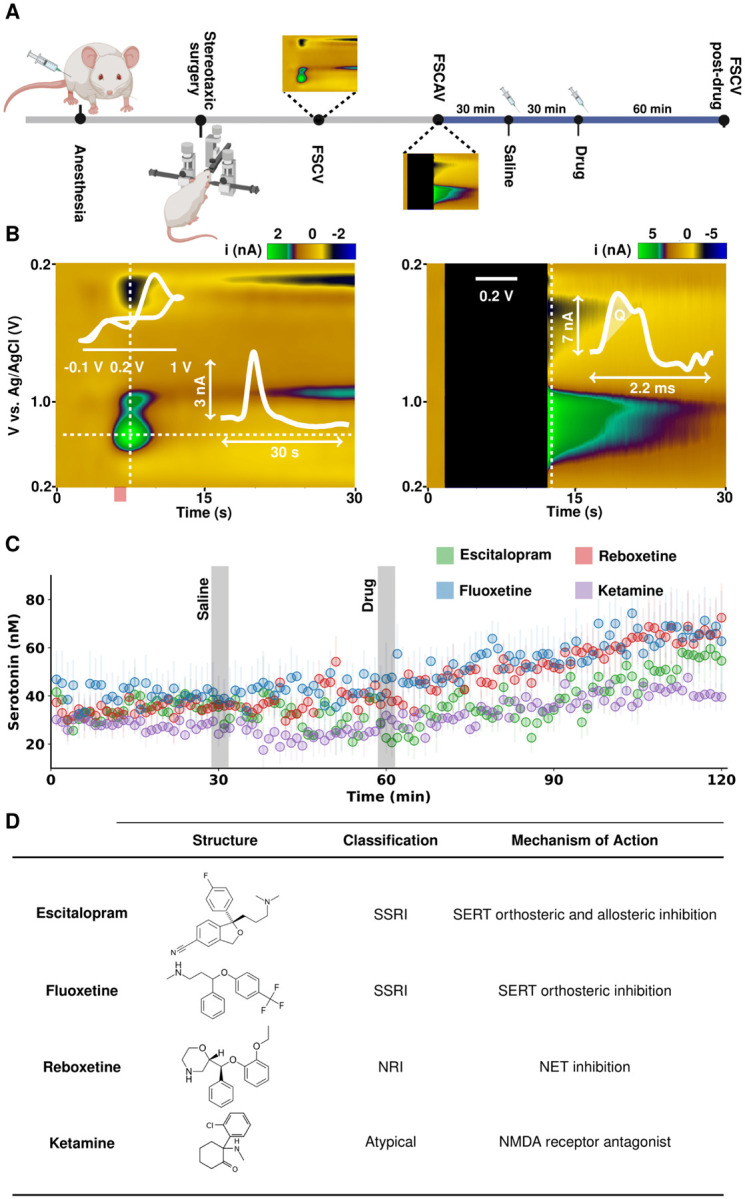
(A) Illustration of experimental paradigm for *in vivo* voltammetry for serotonin measurements with FSCV and FSCAV. (B) Representative *in vivo* serotonin color plots from FSCV (left) and FSCAV (right) signals. The FSCV plot has an inset cyclic voltammogram (top) and current vs. time trace (bottom). The FSCAV plot has an inset cyclic voltammogram where the serotonin peak is integrated for its charge (Q). (C) Effect of antidepressant therapies on ambient serotonin levels. After 30 minutes of baseline measurements, 5 mL kg^−1^ saline was administered (*i.p*.). Then, 20 mg kg^−1^ fluoxetine (blue), 10 mg kg^−1^ escitalopram (green), 20 mg kg^−1^ reboxetine (red) or 10 mg kg^−1^ ketamine (purple) was administered *i.p*. 30 minutes after saline. Each trace shows the average (n = 5 mice from a mixed sex cohort) and associated standard error of the mean (SEM) as error bars. (D) Table outlining the antidepressants used in this study, their structure, classification as an antidepressant and proposed mechanisms of action.

Figure 1A shows the experimental paradigm for serotonin voltammetry. Animals were anesthetized, underwent stereotaxic surgery whereby a stimulating electrode was placed in the medial forebrain bundle (MFB) and a carbon fiber microelectrode (CFM) was placed in the CA2 region of the hippocampus. FSCV was used to identify an area where electrically evoked serotonin was present. The experimental mode was then switched to FSCAV to acquire minute-to-minute basal level measurements. After 30 minutes of control files, 5 mL kg^−1^ saline was given intraperitoneally (*i.p*.), and 30 minutes after that, a drug was given (*i.p*.). 60 minutes after drug files were collected, FSCV was reinstated to assess the effect of the drug.

Figure 1B shows representative FSCV and FSCAV color plots, cyclic voltammograms (CV) and current *vs*. time (IT) (for FSCV). The black space in the FSCAV color plot indicates the holding period. Figure 1C shows the ambient serotonin levels after drug challenge with escitalopram, fluoxetine, reboxetine and ketamine. Each trace is an average of 5 animals ± the SEM (error bars). Figure 1D shows the structure and mode of action for each agent. A repeated measures analysis of variance (*ANOVA*) and analysis of covariance (*ANCOVA*) with *post-hoc* comparisons were used to analyze time points and slopes. Saline administration did not have a significant effect with respect to control prior to escitalopram, fluoxetine, reboxetine and ketamine (values in SI). Sixty minutes after drug, escitalopram, fluoxetine, reboxetine and ketamine significantly increase serotonin. The fastest rate of increase was found after escitalopram, which was significantly higher than fluoxetine and ketamine, but not reboxetine.

### Serotonin Reuptake Kinetics Between Different Antidepressants

**Figure 2.**
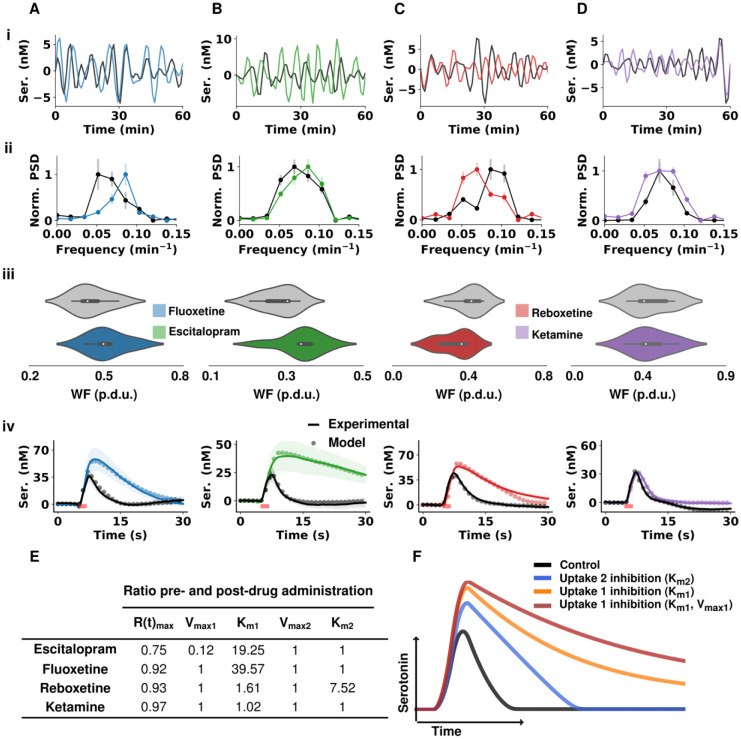
(A) data representing fluoxetine (B) data representing escitalopram (C) data representing reboxetine (D) data representing ketamine (i) Representative FSCAV serotonin (ser.) extracellular oscillations over 60 minutes with control in black and post-drug administration in color. Time series were bandpass filtered (3rd order Butterworth filter with cutoff frequencies of 0.0008 Hz and 0.005 Hz to remove high frequency noise and slow drug effects on the serotonin extracellular levels. (ii) Normalized mean and SEM (n = 5 animals) power spectrum density of control FSCAV data (black) and post-drug data (color). (iii) Violin plots of the distribution of power-weighted frequencies (n = 5 animals for each drug) of the power spectra pre- (gray) and post-drug (color) administration shown in ii. (iv) current *vs*. time profiles from stimulated serotonin release in the CA2 pre- and post-drug administration (n = 5 mice for fluoxetine, reboxetine and ketamine and n = 9 for escitalopram). The solid black traces are from control files and the colored traces are 60 minutes post-drug administration (*i.p*.). Modeled data are laid over their respective traces with dots. The start and end of stimulation is shown with a red bar. (E) Table of M-M terms from modeled data in iv. Each value is the ratio of change post-drug with respect to the control state. (F) Modeled traces with varied M-M terms. Control modeled trace is shown in black (K_m1_ = 2 nM, V_max1_ = 12 nM s^−1^, K_m2_ = 170 nM, V_max2_ = 780 nM s^−1^), competitive Uptake 2 inhibition (K_m2_ is 10-fold the control value) in blue, Uptake 1 competitive inhibition (K_m1_ is 10-fold the control value) in orange and Uptake 1 (K_m1_ is 10-fold and V_max1_ is 50% of the control values) in red.

We performed power spectra density analysis in FSCAV experiments with the 4 antidepressants above. Figure 2A(i) shows a representative example of the filtered FSCAV data collected for 60 minutes before (black) and 60 minutes after fluoxetine (blue), Fig. 2A(ii) is the mean and SEM (n = 5 mice) normalized PSD before drug (black) and after drug (blue), Fig. 2A(iii) is a violin plot showing the distribution of the sum of power-weighted frequencies (WF) from the power spectra. Figure. 2B, C and D show the same data and analysis for escitalopram (green), reboxetine (red) and ketamine (purple). Using the sum of power-weighted frequencies (a measure of displacement of the whole power spectra, see Methods section), we found the weighted frequencies increased after administration of fluoxetine and escitalopram (suggesting Uptake 1 inhibition), decreased after administration of reboxetine (implying Uptake 2 inhibition) and did not change after ketamine administration.

To further test how these agents changed the profile of serotonin reuptake we performed FSCV analysis of evoked serotonin. In Fig. 2(iv) we show control (black) and 60 minutes after drug (color) stimulated hippocampal serotonin release. We found that the maximum amplitude of release and clearance rate significantly increased 60 min after administration of fluoxetine, escitalopram and reboxetine, but not ketamine.

Next, we fit these responses with the M-M model of dual reuptake (Uptake 1 and 2) shown in Equation 1:

(1) $$dC(t)/dt=R(t)(1-A(t))-\alpha \frac{V_{\text{max}1}\cdot C(t)}{K_{m1}+C(t)}-\beta \frac{V_{\text{max}2}\cdot C(t)}{K_{m2}+C(t)}$$

Where *C(t)*, *R(t)* and *A(t)* represent the concentration of the neurotransmitter, evoked release rate and autoreceptor control at time t, respectively. *V*_*max*_ and *K*_*m*_ are M-M parameters and *α* and *β* are the rates of Uptake 1 and Uptake 2. In this model, Uptake 1 represents a high-affinity, low-capacity system (serotonin transporters (SERTs)) and Uptake 2 is a low-affinity, high-capacity system (norepinephrine, dopamine, organic cation transporters and plasma membrane monoamine transporters (NETs, DATs and OCTs and PMATs)). The results of the modeling (shown as ratio of change with respect to control) are in the table in Fig. 2E. Figure 2F is a synthesized response where hypothetical scenarios are modeled. These scenarios are Uptake 2 inhibition *via* K_m2_ (blue), Uptake 1 inhibition *via* K_m1_ (orange) and Uptake 1 inhibition (*via* change in both K_m1_ and V_max1_; red). SERT inhibition with fluoxetine follows a typical orthosteric inhibition profile, where a change in K_m1_ (orange curve in Fig. 2F) can easily fit the curve. Uptake inhibition with reboxetine follows mainly Uptake 2 inhibition, where the curve can be modeled by primarily a change in K_m2_ (resembling the blue trace in Fig. 2F). Ketamine does not change the kinetics of the curve. Escitalopram is the most unusual response in that it cannot be fit with a change in K_m1_ (consistent with SERT inhibition). Here we also needed to substantially alter V_max1_ to fit the curve (red curve in Fig. 2F). Competitive uptake inhibition should not ordinarily change V_max_ therefore we found it interesting to study escitalopram more thoroughly *via* detailed dose response experiments.

### A Temporo-Dose Response for Escitalopram

**Figure 3.**
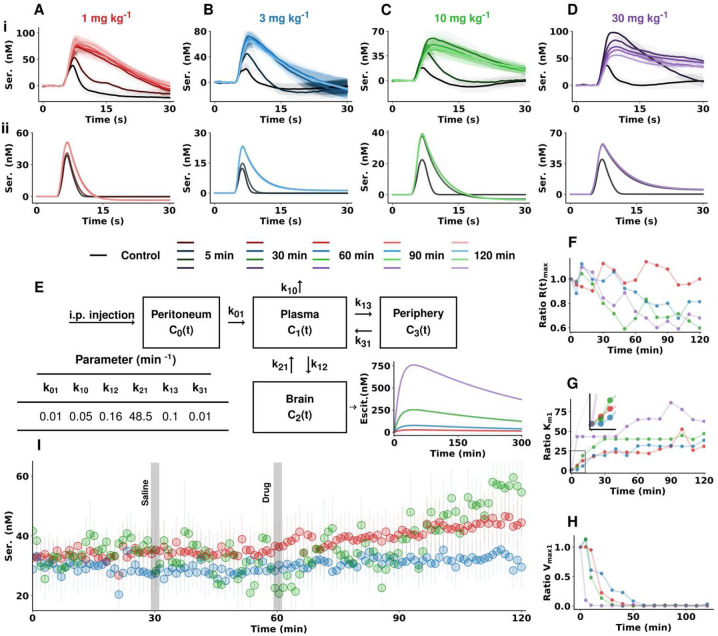
Escitalopram data for (A) 1 mg kg^−1^ (B) 3 mg kg^−1^ (C) 10 mg kg^−1^ (D) 30 mg kg^−1^. (i) Evoked serotonin concentration vs. time traces from the CA2 region of the hippocampus of female mice before and after escitalopram (escit) administration (n = 4 animals each dose, mean ± SEM). (ii) Modeled serotonin concentration vs. time traces from the control data and after changing K_m1_ based on a simulated concentration of escitalopram in the hippocampus with time according to the pharmacokinetic model depicted in (E) (see Methods section for a detailed description of the model). Compartment rate constants are obtained from literature of previous pharmacokinetic models of escitalopram in mice(11) (F-H) Modeled changes in maximum release rate of serotonin (F), K_m1_ (G) and V_max1_ (H) for each dose and over time after drug injection. (I) FSCAV recordings of absolute concentrations of extracellular serotonin following the same experimental paradigm as described in Figure 1 for 3 different doses of escitalopram (mixed sex cohorts, n = 5 animals for 1 mg kg^−1^ dose, n = 7 animals for 3 mg kg^−1^ and 10 mg kg^−1^ doses).

To evaluate the effects of different doses of escitalopram on serotonin, we administered 4 different doses to cohorts of mice and performed a time after drug analysis for each dose (1–30 mg kg^−1^ in Fig. 3A–D(i)). The data is from female mice, however male mice respond similarly and are shown in the supplementary information (**Fig. S1**). The control (before drug) response is shown in black for all doses and then shown 5, 30, 60, 90 and 120 minutes after escitalopram. Figure 3E depicts our pharmacokinetic model, the four-compartment model (FCM), used to obtain an estimation of escitalopram concentration in a mouse brain. In this model, we simulate the physiological path of an acute *i.p*. injection of escitalopram, from 4 different compartments in the body with different concentrations of the drug: the peritoneum (C_0_(t)), plasma (C_1_(t)), brain extracellular space (C_2_(t)) and periphery (C_3_(t)). The arrows of the model depict interchange of escitalopram between compartments. The rate constants, k, determine the speed of escitalopram interchange (nM min^−1^) from one compartment to another, or to secretion (k_10_). The model considers the partial bioavailability of the drug after peritoneal injection, the percentage of protein binding (*e.g.*, albumin) to the drug in plasma and the retention of escitalopram in the brain due to binding to SERTs. Solving the system of equations provides IT traces for escitalopram for each compartment.

Figure 3A–D(ii) are the results of the theoretical temporo-dose response where K_m1_ is altered as per theoretical uptake inhibition(10) of SERTs from escitalopram, while the rest of the release and uptake parameters are kept constant with respect to control. In these theoretical curves, as the dose increases, both maximum amplitude and t_1/2_ of reuptake increase. In terms of the temporal response, for the 1 mg kg^−1^ dose and 5 minutes after drug the response is not meaningfully different from control, and the 30-, 60-, 90- and 120-minute responses are not substantially different from each other, since the modeled concentration of escitalopram (Fig. 3E) does not substantially change. For the 3 mg kg^−1^ dose, this behavior is repeated. For the 10 and 30 mg kg^−1^ doses, the 5-minute responses are not different from the later time points due to higher doses increasing escitalopram in the brain more rapidly. Figure 3F–H shows the ratio changes of maximum release, K_m1_ and V_max1_. In Fig. 3F, the ratio of release amplitude does not substantially change with time for the 1 mg kg^−1^ dose. For all other doses it falls with time. For all doses, the K_m1_ increases with time. A finding of interest is that while at 1 mg kg^−1^ after 5 min the K_m1_ increases, but for 3 mg kg^−1^ at 5 minutes K_m1_ does not increase (inset). In general, V_max1_ decreases with time for all doses. A finding of interest here is that for 3 and 10 mg kg^−1^, V_max1_ increases 5 min after drug administration. Figure 3I is a comparison of the effects on the basal serotonin levels in separate cohorts of mice for 1, 3 and 10 mg kg^−1^. We chose to compare these three doses to gather more information on the similarity between K_m1_ of the 3 and 10 mg kg^−1^ doses. After a control period of 30 minutes, a 5 mL kg^−1^ saline *i.p*. injection was given, and files were collected for another 30 minutes. After this, the drug was given, and the serotonin levels were measured for a further 60 minutes. Saline did not have a significant impact on the measured extracellular levels of serotonin prior to a dose of 1 mg kg^−1^, 3 mg kg^−1^ and 10 mg kg^−1^. At the end of this period, serotonin increased when animals were given 1 mg kg^−1^, 3 mg kg^−1^ and 10 mg kg^−1^ doses. The fastest rate of increase of extracellular serotonin was found to be after administration of a dose of 10 mg kg^−1^, which was significantly higher than after a dose of 3 mg kg^−1^ and 1 mg kg^−1^. An interesting finding is that 1 mg kg^−1^ causes a higher rate of serotonin increase *vs*. 3 mg kg^−1^.

### SERT Expression/Function in Cellular Model of In Vivo-like Serotonergic Transmission

**Figure 4.**
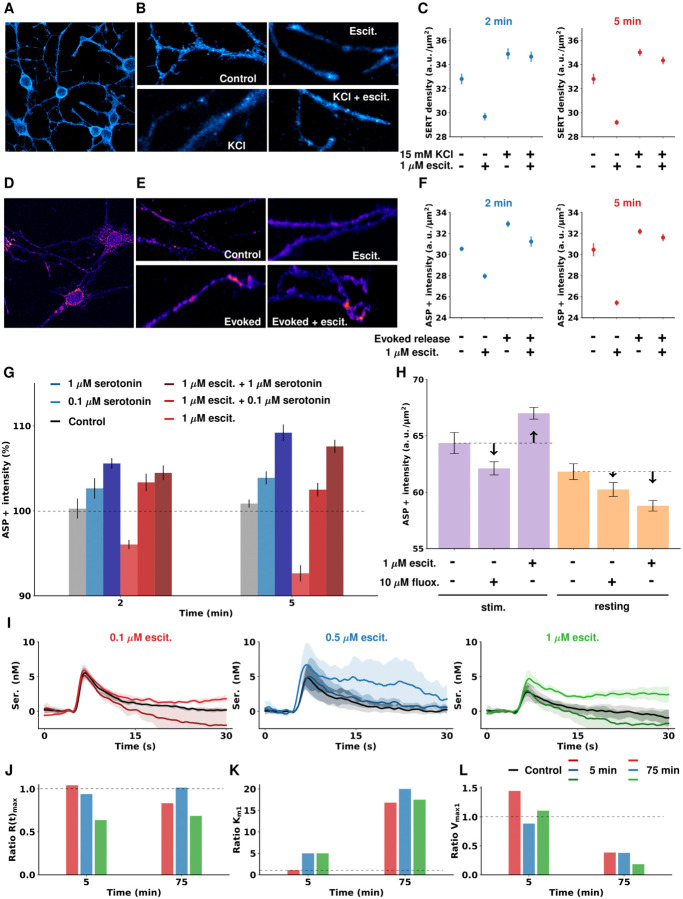
(A) Immunofluorescence of cell surface-located SERT molecules. Fluorescence signals for SERT antibodies targeted against the second extracellular loop localized to neurites and cell bodies of 1C11^5-HT^. (B) Representative regions of interest used for quantification of SERT cell surface density in absence and presence of 1 μM escitalopram with and without high potassium-induced serotonin release 2 min after administration. (C) Average and SEM (n = 80) calculated SERT density in cell cultures 2 and 5 minutes after 15 mM KCl and/or 1 μM escitalopram administration. High potassium-evoked serotonin release results in elevated SERT cell surface densities and diminishes acute escitalopram-induced SERT internalization. (D) The fluorescent SERT substrate ASP^+^ is taken up by 1C11^5-HT^ cells and localizes to neurites and cell bodies. (E) Representative regions of interest used for quantification of SERT uptake in absence and presence of 1 μM escitalopram with (evoked) and without (resting) electrical stimulation for serotonin release. (F) Average and SEM (n = 37) ASP^+^ uptake measurements in cell cultures after evoked release of serotonin and/or 1 μM escitalopram administration. Electrically evoked serotonin release results in increased uptake of fluorescent SERT substrate 2 and 5 minutes after stimulation while escitalopram treatment on its own diminishes SERT-dependent uptake. (Note: more uptake reflects more functional SERT molecules; less uptake means either internalized or inhibited SERT molecules). (G) Average and SEM (n = 37) ASP^+^ uptake under administration of serotonin (0.1 μM or 1 μM), escitalopram (1 μM) or both. (H) Mean and SEM (n = 50) comparison of ASP^+^ uptake by 1C11^5-HT^ exposed to either 1 μM escitalopram or 10 μM fluoxetine (fluox.) during rest or 5 minutes after electrical stimulation. Compared to escitalopram treatment, fluoxetine treatment did not significantly affect ASP^+^ uptake. (I) Mean and SEM (n = 3 cell dishes) evoked voltammetric recordings of serotonin release in control state and 5 and 75 minutes after escitalopram administration (0.1 μM, 0.5 μM, 1 μM). (J, K, L) Fitted Michaelis-Menten parameters (R(t)_max_, K_m1_ and V_max1_) of the concentration *vs*. time traces shown in panel I. Dashed lines represent a ratio of 1 in G and J-L and the control levels in H.

We studied SERT expression and function in a mouse model of serotonergic transmission. Figure 4A shows immunofluorescence of cell surface-located SERTs(12, 13). Figure 4B are representative regions of interest where SERTs were quantified at rest, 2 minutes after potassium (K^+^) stimulation, 1 μM escitalopram, and with both K^+^ and escitalopram. Figure 4C shows that escitalopram caused significant reductions in SERT expression after 2 and 5 minutes with respect to control, as seen previously for longer SSRI exposure (> 2 hrs)(14). K^+^ stimulation and a combination of K^+^ and escitalopram caused an increase in SERT expression after 2 and 5 minutes (significant at 5 minutes). These data show that K^+^ stimulation and a combination of K^+^ stimulation and escitalopram increase surface SERT density.

Figure 4D shows that the fluorescent SERT substrate ASP^+^ is taken up by the cells and localized to neurites and cell bodies. Figure 4E shows example regions of interest used for quantification of SERT uptake at rest, with electrical stimulation, 2 minutes after 1 μM escitalopram, and with electrical stimulation and escitalopram. Figure 4F shows that ASP^+^ uptake diminished 2 minutes and 5 minutes after escitalopram (significant). Electrical stimulation alone and a combination of electrical stimulation and escitalopram increased ASP^+^ uptake after 2 and 5 minutes (near significant). These data show that, in synergy with increasing SERT density, electrical stimulation and escitalopram increase SERT activity.

We next showed that an acute application of only serotonin (0.1 μM and 1 μM) resulted in increased ASP^+^ uptake after 2 minutes and 5 minutes (Fig. 4G). Adding escitalopram to the excess serotonin model was not able to reverse the increase in ASP^+^ uptake. This increased reuptake behavior did not extend to another SSRI, fluoxetine. Figure 4H is a comparison of ASP^+^ uptake in the presence of 1 μM escitalopram or 10 μM fluoxetine with the cells at rest or 5 minutes after electrical stimulation. Without stimulation, fluoxetine did not significantly affect ASP^+^ uptake, while escitalopram significantly decreased ASP^+^ uptake. Fluoxetine + electrical stimulation did not significantly affect ASP^+^ uptake. These data show that this phenomenon of increased ASP^+^ reuptake is limited to escitalopram and not fluoxetine.

Figure 4I are voltammetric recordings in the cells 5 and 75 minutes after the administration of escitalopram at 3 different doses (0.1 μM, 0.5 μM and 1 μM). The average traces were fitted with the two-reuptake M-M equation shown above. Figure 4J–L shows the fitted ratio values with respect to the control state of R(t)_max_, K_m1_ and V_max1_, 5 and 75 minutes after the administration of escitalopram in the abovementioned doses. Interestingly, shortly after escitalopram (5 minutes) we see an increase in the rate of serotonin reuptake for 0.1 μM and 1 μM, and no substantial effect for the 0.5 μM dose. The reuptake rate slows progressively for all doses up to 75 minutes after this. These data provide chemical evidence that, as a result of escitalopram administration, serotonin can be reuptaken at a faster rate (depending on dose and time) due to increased SERT expression.

### Histamine Mediates Ketamine’s Effects on Ambient Serotonin

**Figure 5.**
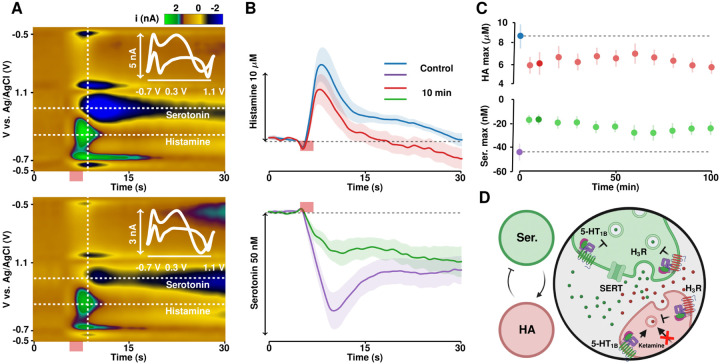
(A) Representative color plots for simultaneous histamine and serotonin monitoring pre-ketamine administration (top) and 40 minutes post-ketamine administration (bottom) with the associated cyclic voltammograms interlaid. (B) IT curves for histamine (top) and serotonin (bottom) pre-and post-ketamine. (C) Evoked histamine amplitude (top) and serotonin inhibition (bottom) over time. (D) Suggested mechanism of action of ketamine on histamine and serotonin.

In this experimental paradigm, we administered ketamine (10 mg kg^−1^ *i.p.)* and monitored the changes in evoked histamine and serotonin dynamics over 100 min in the posterior hypothalamus (PH) of mice. Figure 5A shows representative examples of control histamine and serotonin color plots in the PH (top) and 40 min after administration of ketamine (bottom). Interpretation of these histamine/serotonin color plots can be found elsewhere in great detail(15). Briefly, evoked histamine release inhibits serotonin firing (*via* H3 receptors), the histamine and serotonin events are denoted in the color plot (Fig. 5A) and the concentration *vs*. time profiles (n = 5 animals, mean ± SEM) are shown in Fig 5B. Ketamine induced a rapid decrease in the maximum amplitude of histamine release, with no significant effect on clearance rate was found 10 min after injection. Overall, we found significantly less inhibition of serotonin 10 min after drug injection. Figure 5C shows the average and SEM of the maximum amplitude of histamine (top) and serotonin inhibition amplitude (bottom) over time. The effects of ketamine are sustained 100 min after injection.

## Discussion

### Antidepressants with Differing Modes of Action Increase Basal Extracellular Serotonin

Despite the high prevalence of depression and decades of research into antidepressant drug discovery, this disorder remains difficult to effectively treat. Little progress has been made towards improving the clinical efficacy of antidepressants since the 1950s where the first of these agents (monoamine oxidase inhibitors (MAOIs)) were discovered, up to the modern day where “atypical” treatments such as ketamine are currently being explored. Importantly, there is now heated discussion about whether clinical responses to antidepressants (historical or modern) are even considered significantly meaningful with respect to placebo, exercise or psychotherapy (2, 3, 16).

The overarching issue is that there are no reliable pre-clinical screening tools for antidepressants. Traditionally, potential antidepressant efficacy was screened using the forced swim test (FST) in rodents(17). The length of time it took for animals to enter a learned helplessness state was improved by acute injections of potential antidepressants(18). While many agents created this behavioral shift in rodents, they failed at clinical trials(19).

Moreover, few animal models have adequately captured behavioral changes in response to a chronic antidepressant regime (that humans undergo)(20–25). As such, pharmaceutical companies dramatically toned down their antidepressant drug discovery efforts over the last 15 years and the larger research community, more recently, is raising serious concerns about the validity of the FST to accurately reflect depression phenotypes (26–29).

It is nearly impossible to systematically develop more effective antidepressants without a working hypothesis of the chemical basis of depression. A screening process would ideally measure a biomarker of disease and gauge the response of this biomarker to potential therapeutics. Because of the monoamine hypothesis of depression, serotonin has long been speculated as a biomarker; however, because serotonin is difficult to measure *in vivo* and SSRIs have limited clinical efficacy, serotonin has become unpopular as a depression target in recent years and the research community have focused on new therapies outside of serotonin (such as NRIs and ketamine). However, despite the evident mode of action of these different antidepressants, there is still quite some speculation that these agents target serotonin(6–9). Thus, we sought to assess the effects of SSRIs, an NRI and ketamine on brain serotonin with ultra-sensitive, niche voltammetric tools.

Mechanistically, SSRIs block serotonin transporters orthosterically (escitalopram and fluoxetine) and allosterically (escitalopram)(30, 31). NRIs inhibit norepinephrine transporters(32) and ketamine is a non-competitive antagonist of NMDA receptors(33). We administered these drugs as large, acute, therapeutically equivalent doses because this is where the most robust behavioral shifts were previously found for FST(34–36). We found, that despite their different modes of action, acute administration of each antidepressant caused a significant increase in basal levels of extracellular serotonin.

For SSRIs, the source of the increased serotonin levels is clear since inhibition of the serotonin transporter should allow more serotonin to accumulate in the extracellular space. Our previous work(37–40) and others(41, 42) support this mechanism. For reboxetine the source is less clear since this agent targets NETs with high affinity over the SERTs(43, 44) and previous microdialysis studies did not find serotonin to change after this drug(45). Similarly, for ketamine a mechanism for increased serotonin is not obvious although there are reports in the literature that ketamine may increase extracellular serotonin(46). In the next sections, we explore in-depth our experimental findings of the mechanisms that contribute to increased serotonin for these agents.

### Antidepressants that Target Uptake 1 and 2 Increase Basal Serotonin

Monoamine uptake inhibition has been the frontline pharmaceutical strategy for depression. The classical view that membrane bound transporters are only responsible for clearing their namesake substrate (*i.e.*, SERTs for serotonin) has been significantly refined in recent years. We now know that monoamine transporters are ‘promiscuous’ and reuptake each other’s substrates(47, 48). Serotonin is known to be taken up *via* two systems: Uptake 1 and Uptake 2(49). Briefly, as described above, Uptake 1 is uptake *via* SERTs, a high-affinity, low-capacity system, whereas Uptake 2 is transport *via* a combination of the other monoamine transporters: DATs, NETs, OCTs and PMATs, which is a low-affinity, but high-capacity system(50).

In previous work, we discovered that ambient extracellular serotonin in the hippocampus slowly oscillates with a period of around 6–10 minutes and showed that pharmacological SERT inhibition changed the frequency of the oscillations(51). We attributed the change in that frequency to Uptake 1 inhibition, illustrating the validity of oscillation analysis to accurately capture which transporters are activated. For example, when SERTs are blocked, the high-capacity Uptake 2 system should dominate, thus the cycling speed should be faster than in a system controlled only by SERTs (*i.e*., lower capacity). Conversely when NETs are blocked, the Uptake 1 system will dominate, therefore the cycling frequency should slow compared to an Uptake 2 (high-capacity) system.

Here we repeated the oscillation analysis for escitalopram and the other 3 agents. We found, in accord with our reasoning above, that escitalopram and fluoxetine both increased the frequency of basal oscillations, pointing to Uptake 1 inhibition, reboxetine decreased these oscillations and ketamine had no effect. We believe that this is strong evidence showing that reboxetine plays a significant role in serotonin reuptake *via* Uptake 2 inhibition. Reboxetine effects on serotonin reuptake have been previously reported but were shown to be less significant compared to effects on norepinephrine reuptake(52). To test this more formally, we performed FSCV analysis pre- and post-drug in the same mice. For FSCV, serotonin is electrically evoked *via* MFB stimulation, and we have found it can capture dynamic changes in reuptake(40, 53). Indeed, we found that escitalopram, fluoxetine and reboxetine all decreased the rate of serotonin reuptake, while ketamine had no effect.

To analyze the FSCV curves, we next applied M-M analysis with a previously established model from our lab(40) that incorporated both reuptake mechanisms with 2 sets of K_m_ and V_max_. Using that model, we found that by only altering K_m1_ to fit the uptake curve, SERT inhibition with fluoxetine followed a classical orthosteric inhibition profile. Whereas serotonin uptake following reboxetine exhibited mainly Uptake 2 inhibition, as primarily K_m2_ was altered to fit the curve. In conjunction with the decrease in oscillatory frequency following reboxetine administration, these data clearly show that reboxetine increases basal serotonin levels *via* Uptake 2 inhibition.

A surprising finding of these analyses was that we could not fit the escitalopram response with the classical K_m1_ inhibition model but also had to substantially alter V_max1_ to fit the curve. Competitive uptake inhibition should not alter V_max1_, however escitalopram is an unusual SSRI in that it binds the SERTs both orthosterically and allosterically(54). As it has been also postulated to be the most clinically efficacious SSRI(55), we next focused on understanding escitalopram’s unusual reuptake kinetic profile.

### Dynamic Escitalopram-Induced Expression of Serotonin Transporters

From our M-M analysis of the FSCV signals, we found that we could not model the response to escitalopram with a classic orthosteric Uptake 1 inhibition and had to vary V_max1_ to fit the curve. This implies that the effects of escitalopram on SERTs transcend simple competitive uptake inhibition. This finding may be significant since there is ample evidence in the literature implying escitalopram is the most clinically efficacious antidepressant(55, 56). Some attribute this effect to the fact that escitalopram binds the SERTs both orthosterically and allosterically(30, 54). Thus, to further explore the effects of escitalopram on serotonin signaling, we investigated an escitalopram dose response *via* a set of coordinated experiments and theoretical modeling.

In separate cohorts of mice, we gave 4 different doses (n = 4 for each dose) of escitalopram and collected FSCV files before giving the drug, 5 minutes post-drug and then every 10 minutes post-drug for 120 minutes. In parallel, we investigated how the theoretical response to these doses should look like under pure competitive inhibition. To this end, we created a pharmacokinetic framework model, that we coin the four-compartment model (FCM), to predict the distribution of escitalopram in the body after an *i.p*. injection. The concentration of escitalopram at a specific dose and time post-injection in the brain was used to predict the new affinity of SERTs for serotonin (apparent K_m1_, see Materials and Methods section). The control experimental traces were modeled using our Uptake 1 and 2 reuptake M-M model. To predict changes in reuptake produced by the competitive inhibition of escitalopram, K_m1_ was replaced with the new apparent K_m1_. Surprisingly, we found that none of the FCM responses mirrored the experimental FSCV signals and present 3 major differences below:

#### Evoked Serotonin Amplitudes were Higher than the FCM Predicted

For all doses, the FCM predicted maximum amplitudes after drug lower than found in experiments. The experimental responses were well fit by decreasing V_max_. A decrease in V_max_ could occur with a decreased number of transporters, which decreases the reuptake capacity.

#### 10 Minutes After Each Dose, Reuptake Rates were Slower than Theory Predicted

10 minutes after each dose, all experimental reuptake rates were slower than in the predicted models. After 10 minutes the curves could, again, only be fit by reducing V_max_ (as above, V_max_ can mean fewer SERTs(57)). Rapid, SRRI-induced SERT internalization has previously been reported (58). This internalization mechanism is not believed to be mediated by allosteric binding (54). SERT trafficking to and from the membrane is regulated by kinase internal signaling pathways such as protein kinases C (PKC)(59). The mechanism of SSRI-induced SERT internalization is not yet fully defined, but the decrease in SERT surface density is analogous to PKC activation(60), bringing forth PKC activation as a possible mechanism. SERT internalization after chronic SSRI paradigms has also been observed(61).

#### 5 Minutes After the 3 and 10 mg kg^−1^ Doses Reuptake Rates were *Faster* than Control

Finally, experimental reuptake rates 5 minutes after the 3 and 10 mg kg^−1^ doses were *faster* than control, not slower, as predicted by the FCM (and general uptake inhibition theory). This phenomenon is more enigmatic than the previous two. We propose that this effect is likely compensatory (bidding to regulate increasing extracellular serotonin levels), threshold based (not present for the 1mg kg^−1^ dose), involves autoreceptors and runs in parallel to SERT internalization.

The clearance slopes for the 3 mg kg^−1^ response are not different (and sometimes faster) than the 1 mg kg^−1^ response. These slopes should be slower according to the FCM and general theory (*i.e*. more drug equals more effect). This finding implies that a compensation mechanism has been activated to counteract the increasing dose. More evidence for this comes from an FSCAV dose response for basal levels where the 3 mg kg^−1^ response is blunted *vs*. the 1 mg kg^−1^, showing compensation that is clearly overridden by 10 mg kg^−1^.

In trying to find a mechanistic source of this compensation, 2 studies guided us. Firstly, in an elegant study, Blakely and colleagues showed a rapid and clear *increase* in serotonin reuptake activity as a result of immune system activation produced by peripheral LPS in mice (62, 63). Secondly, we were inspired by the fact that SERTs traffic rapidly across the membrane, a phenomenon that can be experimentally modeled in murine cells(64, 65). Therefore, we decided to study serotonin reuptake optically and with voltammetry in a cellular model of serotoninergic neurons.

Knowing that escitalopram induces SERT internalization after several hours, we asked what happens after a few minutes (to mimic our dynamic *in vivo* response at 5 minutes). We found, in accord with the previous studies of longer time exposure (hours), that in these cells, after 5 minutes of escitalopram alone there were fewer SERTs on the cell surface and less ASP^+^ reuptake. However, when we stimulated the cells electrically, with K^+^ or serotonin, there were *more* serotonin transporters on the cell surface and *more* ASP^+^ reuptake. While the stimulations are physiologically high, the resting neurons in this model are also aphysiological in their limited ambient activity. The physiological reality *in vivo* is likely somewhere in the middle of resting and stimulated. Nonetheless the stimulated cells mimic our *in vivo* experiment and thus are a good parallel. Additionally, in accord with our *in vivo* data, this SERT overexpression/internalization(58) phenomenon is not induced by fluoxetine. A set of voltammetric experiments in the cell model supported all these microscopic findings of dynamic escitalopram-induced increase and decreased SERT expression.

Therefore, we believe that there are parallel SERT shuttling mechanisms, one of which (overexpression) is clearly compensatory, that take effect after acute escitalopram (with the unusual property to elicit both) and that we’re able to capture the first because of the rapidity of our voltammetric method. The natural question here is do these mechanisms contribute to the clinical regime where antidepressants must be taken for weeks for clinical efficacy?(66) Many conjecture that SERT internalization is indeed an endpoint of antidepressant efficacy(14), we add here that perhaps the intervening weeks where patients often feel worse before they feel better may be underpinned by SERT overexpression. This should be addressed by the community in future work on this topic.

Up until now, we have proposed different mechanisms by which fluoxetine, reboxetine and escitalopram raise serotonin extracellular levels. We next move on to investigate the processes by which ketamine has an effect on serotonin levels.

### Ketamine Exerts its Effects on Serotonin via Histamine

Ketamine is a potent non-competitive NMDA receptor antagonist, conventionally used in high doses to induce anesthesia(33). The use of acute, subanesthetic doses of ketamine to treat depressive symptoms has drastically grown in popularity for patients that are treatment-resistant (67). However, there are grave concerns about the long-term safety of this agent, since it is a controlled substance with well-known and potent adverse side effects(68, 69). As the exact mechanism is unknown, several theories have been put forth on the potential antidepressant action of ketamine. Ketamine has been shown to activate the mammalian target of rapamycin (mTOR) pathway, which promotes synaptogenesis in the prefrontal cortex and hippocampus(70, 71). This is believed to work *via* antagonism of NMDA receptors on GABA interneurons, which subsequently disinhibit glutamatergic neurons *via* AMPA receptor activation(71). This effect has been shown to compensate for reduced neurotrophic factor expression under stress and in cases of depression(72). This theory of synaptic plasticity and neurogenesis does not exclude the monoamine hypothesis, since SSRIs have also been shown to increase neurotrophic factor expression, which is thought to be partially induced by an increase in extracellular serotonin levels(73). Additionally, serotonin analogues such as psychedelics have been shown to promote dendritic growth *via* activation of serotonin receptors(74, 75). A parallel idea is that ketamine is an anti-inflammatory agent, as it has also been shown to attenuate proinflammatory cytokine levels during inflammation(76). Inflammation is almost exclusively synonymous with depression(77). Recently, we demonstrated *in vivo* that stress- and LPS-induced inflammation deplete extracellular serotonin levels in the brain(78). The mechanism for this, in part, is that inflammation-induced histamine inhibits serotonin *via* inhibitory H_3_ heteroreceptors on serotonin neurons(79). We therefore decided to probe whether histaminergic modulation of serotonin is the missing link between the rise in extracellular serotonin levels after acute administration of ketamine. In the PH of mice, we saw a significant and sustained decrease in the maximum amplitude of electrically evoked histamine release 10 minutes after an acute injection of ketamine (20 mg kg^−1^), and as a consequence, a disinhibition of serotonin release through H_3_ receptors on serotonin terminals. It is well-known that ketamine binds to and inhibits NMDA receptors on GABA interneurons(80) (thereby disinhibiting glutamate signaling). Given that histamine neurons are also known to have NMDA receptors(81), it is likely that ketamine inhibits histamine neurons *via* the NMDA receptors.

In summary, we used *in vivo* FSCV and FSCAV to measure hippocampal serotonin dynamics in mice and studied the effects of 4 different antidepressants: two SSRIs (fluoxetine and escitalopram), an NRI (reboxetine) and ketamine. We found that pseudo-equivalent doses of these drugs similarly increased ambient serotonin levels, despite their differing pharmacodynamics. Next, we took in-depth experimental and analysis approaches to understanding how these drugs modulated serotonin. Through M-M analysis, we found that changes in serotonin after SSRIs and reboxetine were due to differences in Uptake 1 and Uptake 2 mediation of serotonin reuptake and teased out critical differences between escitalopram and fluoxetine. In a cellular model of serotonergic transmission, we showed that escitalopram quickly induced SERT overexpression. Finally, we showed that ketamine modulated serotonin levels *via* the histamine system. Although these drugs have different pharmacodynamics, they have in common direct or indirect effects on serotonin (mechanisms illustrated in **Figure S2**). This finding signifies serotonin may harmonize the different theories of depression and that the community should consider how different antidepressants affect serotonin when looking at future drug design.

## Significance

Antidepressants with different modes of action all increase extracellular serotonin *via* direct and indirect pathways. Unlike fluoxetine and reboxetine, escitalopram rapidly induces serotonin transporter overexpression and internalization, a unique action that is possibly a contributing factor to its supposed superior clinical efficacy. Ketamine increases serotonin levels *via* disinhibition of serotonin through histamine. This work demonstrates that serotonin, when measured directly, may indicate potential antidepressant activity.

## Materials and Methods

### Mice

C57BL/6J mice (Jackson Laboratory, Bar Harbor, ME, USA) were group housed (age: 6–12 weeks, weight: 18–30 g), with *ad libitum* access to food and water, and kept on a 12 h light/dark cycle (lights on at 7:00 AM, lights off at 7:00 PM). A mixed cohort of male and female mice were used to study the effects of different antidepressants. For the escitalopram dose-response study, male and female mice responses were segregated (Figure 2 shows female responses and male responses are in the supplementary information). All mouse experiments presented in this work were performed according to National Institutes of Health (NIH) guidelines and complied with the University of South Carolina Institutional Animal Care and Use Committee under an approved protocol.

### Cell Cultures

1C11^5-HT^ cell culture was performed as described previously(82). Briefly, undifferentiated 1C11 cells were kept on 100 mm plates (Sarstedt) in DMEM Glutamax with 10% fetal bovine serum, 1% non-essential amino acids, 1% penicillin/streptomycin, and 1% L-glutamine (all media components by Life Technologies) at 37^°C^ and 5% CO_2_. For differentiation to serotonin neuron-like cells (1C11^5-HT^) 10.000 cells were transferred to 8-well imaging slides (Ibidi). Then, culture medium was supplemented with 1 mM dibutyryl cAMP and 0.05% cyclohexanecarboxylic acid for 4 days (media supplements by Sigma Aldrich). Treatment conditions: For determination of SERT cell surface density, medium was supplemented with 15 mM KCl and/or 1 μM escitalopram. For ASP^+^ live cell imaging, cells were transferred to FSCV buffer containing 50 μM ASP and/or 1 μM escitalopram and/or serotonin (0.1 or 1 μM). Electrical stimulation was applied after perfusing the imaging slides with ASP^+^-containing FSCV buffer. Dye-free buffer was applied 1 min after electrical stimulation before image acquisition.

### Solutions

Ketamine hydrochloride (Vet One, MWI Animal Health, Boise, ID, USA), fluoxetine hydrochloride (Sigma Aldrich, St. Louis, MO, USA) and reboxetine mesylate hydrate (Sigma Aldrich, St. Louis, MO, USA), were individually dissolved in sterile saline (0.9% NaCl solution, Hospira, Mountainside Medical Equipment, Marcy, NY, USA) and administered *via* intraperitoneal injection (i.p.) at 10 mg kg^−1^ and a volume of 5 mL kg^−1^ body weight. Escitalopram oxalate (Sigma Aldrich, St. Louis, MO, USA) was prepared and administered following the same method at the following doses: 1 mg kg^−1^, 3 mg kg^−1^, 10 mg kg^−1^ and 30 mg kg^−1^. Urethane (Sigma Aldrich, St. Louis, MO, USA) was dissolved in sterile saline at 25% w/v and administered at 7 μLg^−1^ mouse body weight for surgical anesthesia. Post-calibration solutions for FSCAV were prepared by dissolving serotonin hydrochloride (Sigma–Aldrich Co., St. Louis, MO, United States) in Tris buffer to make solution concentrations of 10, 25, 50, and 100 nM. Tris buffer consisted of: 15 mM H_2_NC(CH_2_OH)_2_•HCl, 140 mM NaCl, 3.25 mM KCl, 1.2 mM CaCl_2_, 1.25 mM NaH_2_PO_4_•H_2_O, 1.2 mM MgCl_2_, and 2.0 mM Na_2_SO_4_ (Sigma–Aldrich Co., St. Louis, MO, United States) in deionized water; the pH adjusted to 7.4 (± 0.03).

### Electrode Fabrication

Carbon Fiber Microelectrodes (CFMs) were made individually by aspirating a single carbon fiber (Goodfellow Corporation, PA, USA) into a 0.6 mm × 0.4 mm glass capillary (A-M Systems, Inc., Sequim, WA, USA). The capillary was then pulled in a vertical puller (Narishige, Tokyo, Japan) to create a carbon-glass seal. The exposed carbon fiber was then trimmed to 150 ± 5 *μ*m. Liquion (LQ-1105, 5% by weight Nafion^™^ New Castle, DE, USA) was electrodeposited onto the surface of the carbon fiber by submerging the fiber in Nafion^™^ and applying a constant potential of + 1.0 V for 30 s. The electrode was then dried at 70°C for 10 minutes and used after 24 hours.

### Surgical Procedures

Mice were injected with a 25% urethane solution based on a calculation that is dependent on their weight (7 μL/g). Following anesthetic administration, the mouse was placed into a stereotaxic system (David Kopf Instruments, Tujunga, CA, USA) where body temperature was maintained *via* heating pad (Braintree Scientific, Braintree, MA, USA). Three holes were drilled into the skull of the mouse based on coordinates from the mouse brain atlas(83). For serotonin measurements, the working electrode was placed in the CA2 region of the hippocampus (AP: − 2.91, ML: + 3.35, DV: − 2.50), the stimulating electrode (insulated stainless-steel, diameter 0.2 mm, untwisted, Platistics One, Roanoke, VA, USA) was placed in the MFB (AP: − 1.58, ML: + 1.00, DV: − 4.80) and the pseudo-Ag/AgCl reference electrode (made by chloritizing a silver wire in a solution of 1 M HCl for 30 s at 5 V) was placed in the opposite hemisphere of the brain as the working and stimulating electrodes. For histamine and serotonin co-measurements, the working electrode was placed in the PH (AP: −2.45, ML: + 0.50, DV: −5.45 to −5.55). Stimulation was accomplished *via* linear constant current stimulus isolator (NL800A Neurolog, Medical Systems Corp, Great Neck, NY, USA) with the following parameters: 60 Hz, 360 μA each, 2 ms in width and 2 s in length.

### Cell Measurements Procedures

SERT cell surface density was determined as described previously(84): cells were fixed in 1% paraformaldehyde/1x PBS and subsequently incubated in non-permeabilizing blocking solution (BS, 10% horse serum; 0.2% gelatin in 1x PBS; all chemicals Sigma Aldrich). Then, SERT antibodies (Advanced Targeting Systems), diluted 1:250, were applied in BS for 60 min at room temperature. After primary antibody incubation, cells were washed three times in BS, then secondary Alexa Fluor488-conjugated antibodies (Thermo Fisher Scientific), diluted 1:1000, were incubated for 45 min at room temperature. Subsequently, cells were washed three times in 1x PBS and mounted in Dako Fluorescence Mounting Medium (Dako Cytomation). To determine ROIs for image analysis, either Cellmask Deep Red plasma membrane stain (Thermo Fisher Scientific, diluted 1:1000) was applied before fixation or tubulin immunostaining (Abcam, diluted 1:250) was employed after SERT antibody staining. For ASP^+^ live cell image(85), cells were loaded with 50 μM ASP+ in FSCV buffer for 1 min under the treatment conditions mentioned above. Before image acquisition, cells were transferred to ASP^+^-free containing buffer. To determine ROIs, a 1x working solution of Cellmask Deep Red plasma membrane stain was employed (Thermo Fisher Scientific).

Confocal image stacks were acquired using a Leica TCS SP5 Confocal imaging system mounted on a DM IRE2 microscope, equipped with an acusto-optical beam splitter, an argon ion laser (458 nm – 514 nm), a diode-pumped solid-state laser (561 nm), and a helium neon laser (633 nm). Laser lines were used as recommended for the applied dyes. Single sections of a confocal stack were acquired at 0.5 μm steps. Confocal stacks were imported into NIH ImageJ to generate z-projections and quantify the effect of treatment conditions on fluorescence intensities. Using the channels for determination cell boundaries ROIs were defined using the Multi Measure plugin. ROIs were then imported for quantifications into the fluorescence channels used to acquire SERT or ASP+ fluorescence signals. Fluorescence intensities measured using ImageJ were imported into GraphPadPrism software (GraphPad Software Inc., USA) to generate graphs and perform statistical analysis (variance analysis and post hoc-comparison or Student’s t-test).

For FSCV measurements, cover slips with neuronal cell clusters were placed into a 35 mm low wall imaging dish (Ibidi GmbH, Martinsried, Germany) and were covered with a 2.5 mM glucose solution in HEPES buffer. The dish was then placed into a plastic holder covered with aluminum foil connected to the ground acting as a Faraday cage. Carphone fiber microelectrodes and stimulation electrodes were position into the cell dish, and a reference Ag/AgCl electrode (A-M Systems, Sequim, WA) was also placed in the dish under an inverted microscope (Leica DM IL LED, Wetzlar, Germany).

### Hardware and Data Collection

FSCV and FSCAV were performed using a Dagan Potentiostat, (Dagan Corporation, Minneapolis, MN, USA), National Instruments multifunction device USB-6341 (National Instruments, Austin, TX, USA), WCCV 4.0 software (Knowmad Technologies LLC, Tucson, AZ, USA) and a Pine Research headstage (Pine Research Instrumentation, Durham, NC, USA). The “Jackson” waveform was applied to elicit the redox properties of serotonin (0.2 V to 1.0 V to - 0.1 V to 0.2 V, 1000 V s^−1^)(86). For histamine and serotonin co-measurement, the histamine waveform was applied (−0.5 V to −0.7 V to 1.1 V to −0.5 V at 600 V s^−1^)(15). For FSCV data collection, these waveforms were applied at 10 Hz. The MFB was stimulated *in vivo* for FSCV experiments with biphasic pulses applied using a linear constant current stimulus isolator (NL800A Neurolog, Medical Systems Corp, Great Neck, NY, USA) with parameters: frequency of 60 Hz, amplitude of 360 μA each, 2 ms in width, and 2 s of total duration (120 pulses). For cell culture experiments, the electrical stimulation consisted of biphasic pulses with the following parameters: frequency of 60 Hz, amplitude of 250 μA each, 4 ms in width, and 1 s of total duration (60 pulses).

FSCAV consisted of 4 steps. First, the waveform is applied at 100 Hz for 2 seconds to minimize adsorption into the carbon fiber. Second, a constant potential (0.2 V) is then applied for 10 seconds to allow serotonin to preconcentrate in the fiber surface. Third, the waveform is reapplied for 18 seconds to acquire the information-rich voltammograms. Finally, a waiting time of 30 seconds is given until the next file is acquired. Both for FSCV and FSCAV, the initial 10 cyclic voltammograms were averaged and subtracted from the rest of the cycling voltammograms in the color plot.

### Data Processing, Parametric Analysis and Modeling of Electrochemical Data

FSCV files were exported from WCCV software and filtered, calibrated and analyzed using The Analysis Kid(87). Low pass filtering of color plots was performed using a 3^rd^ order 2D Butterworth filter with a cutoff frequency of 37.5 kHz on the x axis and 2.5 Hz on the y axis.

Serotonin and histamine were converted to concentration using calibration factors from previous in vitro flow injection analysis using the Jackson waveform (49.5 μM nA^−1^ for serotonin)(88) and histamine waveform (11 nM nA^−1^ for serotonin and 2.825 μM nA^−1^ for serotonin)(79). The maximum amplitude of release, Amp_max_, was automatically detected using local maxima algorithms. The half-life of the neurotransmitter, t_1/2_, was calculated by fitting an exponential curve, shown in Equation 1, from the maximum amplitude of the release and calculating the time it takes to reach 50% of the maximum concentration of the trace.

(1) $$C(t)=C_{0}\cdot e^{-k\;\dot{t}},\;\;t_{1/2}=\frac{ln(2)}{k}$$

FSCV traces were also fitted using a two-reuptake M-M model of the release and reuptake of serotonin, shown in Equation 2.

(2) $$\frac{d\;C(t)}{dt}=R(t)(1-A(t))-\alpha \frac{V_{\text{max}1}\cdot Ct)}{K_{m1}+C(t)}-\beta \frac{V_{\text{max}2}\cdot Ct)}{K_{m2}+C(t)}$$

Here, R(t) and A(t) are the release rate and autoreceptor occupancy (modulating the release term), C(t) is the FSCV concentration time series, α and β are the coefficients of the two reuptake mechanisms, V_max1_ and K_m1_ are the M-M parameters of the Uptake 1 mechanism (SERTs), V_max2_ and K_m2_ are the M-M parameters of the Uptake 2 mechanism (OCTs, DATs and NETs). The model is semi-automatically fitted to the experimental data using a custom-designed gradient descent algorithm implemented in The Analysis Kid(87), that uses the RMSE as cost function. The differential equation is solved using the Euler method.

FSCAV files were exported from the WCCV software and calibrated in The Analysis Kid. The third CV after the adsorption period was selected to estimate the concentration of serotonin. Charge under the serotonin Faradaic peak (numerical integral) was calculated using the Simpson’s rule. Integration start and end points are automatically detected using local minima algorithms. To minimize the capacitive charge interference, a baseline area (charge below a line between the first and last integration points) is subtracted from the total charge. Calibration was performed using coefficients from electrode-specific linear regressions obtained from *in vitro* recordings, or a standardized neural network trained with a large in vitro dataset from 140 electrodes. More information on the processing methods are extensively explained elsewhere (87, 89).

FSCAV time traces were filtered using a 3^rd^ order bandpass Butterworth filter with cutoff frequencies of 0.0008 Hz and 0.005 Hz to study the fluctuations of serotonin. The low cutoff frequency was chosen to remove from consideration the low frequency drug effects on serotonin basal concentration. The high cutoff frequency removes the noise component of the signal.

Autocorrelation and sliding window correlation (SWC) using Pearson’s correlation were used to confirm the presence of fluctuation patterns in the signals. The autocorrelation function, expressed in Equation 3, was used to calculate the correlation between pairs of samples distanced by a lag (p), where X denotes the mean value across the time series of length N.

(3) $$r_{p}=\frac{\sum_{i=1}^{N-p}\left(X_{i}-\bar{X}\right)\left(X_{i+p}-\bar{X}\right)}{\sum_{i=1}^{N}{\left(X_{i}-\bar{X}\right)}^{2}}$$

SWC was used to study the correlation of fixed-length windows. Equation 4 shows the general formulation of the correlation function between a window of length l starting at time t and another window of the same length distanced by a lag p(51).

(4) $$r_{t,p}=\frac{\sum_{s=t}^{t+l-1}\left(X_{s}-\bar{X_{s}}\right)\left(X_{s+p}-\bar{X_{s+p}}\right)}{\sqrt{\left(\sum_{s=t}^{t+l-1}{\left(X_{s}-\bar{X_{s}}\right)}^{2}\right)\left(\sum_{s=t}^{t+l-1}{\left(X_{s+p}-\bar{X_{s+p}}\right)}^{2}\right)}}$$

Autocorrelation and SWC were calculated for all delays to study the presence of oscillatory patterns in the time series. A window length of 25 minutes was used for SWC. The power spectral density (PSD) of time series was calculated from the fast Fourier transform spectrum using the Welch method(90). Analysis of fluctuation patterns pre- and post-drug administration were performed by comparison of the PSD peak that corresponds to the largest frequency pattern established with the correlation measurements and the spectrum density.

### Compartmental Modeling of Escitalopram

The pharmacokinetic model was coded using Python generic code and solved using the SciPy library. A four compartmental model (compartments: peritoneum, plasma, periphery and brain) of the pharmacokinetics of escitalopram after intraperitoneal injection was built based on prior experiments with mice and rats. A schematic of the model is represented in Figure 3. Equation 5–8 shows the system of equations of the compartmental model.

(5) $$\frac{dC_{0}}{dt}=-k_{01}C_{0}(t)$$

(6) $$\frac{dC_{1}}{dt}=k_{01}C_{0}(t)-\left(k_{10}+k_{12}\right)\left(C_{1}(t)\left(1-P_{B}\right)\right)+k_{21}\left(C_{2}(t)\left(1-SERT_{B}\right)\right)-k_{12}\left(C_{1}(t)\left(1-P_{B}\right)\right)+k_{31}C_{3}(t)$$

(7) $$\frac{dC_{2}}{dt}=k_{12}\left(C_{1}(t)\left(1-P_{B}\right)\right)-k_{21}\left(C_{2}(t)\left(1-SERT_{B}\right)\right)$$

(8) $$\frac{dC_{3}}{dt}=k_{13}\left(C_{1}(t)\left(1-P_{B}\right)\right)-k_{31}C_{3}(t)$$

C_0_(t), C_1_(t), C_2_(t) and C_3_(t) represent the concentration of escitalopram over time in the peritoneum, plasma, brain and periphery of the mouse body, k_01_ and k_10_ are the rate constants of diffusion from the peritoneum to plasma and secretion from plasma, k_12_ and k_21_ are the rate constants between plasma and the brain and k_13_ and k_31_ are the rate constants between plasma and the periphery, P_B_ represent the ratio of escitalopram binding to protein in plasma and SERT_B_ represents the percentage of escitalopram binding to SERT in the brain.

Rate constants used for the simulation are described in Figure 3. P_B_ and SERT_B_ are set to 0.56 and 0.15, respectively(91). Bioavailability of escitalopram was set to 0.80, a previously calculated bioavailability for oral administration(92), since bioavailability of intraperitoneal injection has not been studied, but it is reasonable to assume that would be between oral (0.80) and intravenous (1.00). Mouse weight was set to 20 g, the midpoint between the female mouse weight (~15 g) and male mouse weight (~25 g). Dose and volume of escitalopram solutions were dependent on mouse weight (doses: 1 mg kg^−1^, 3 mg kg^−1^, 10 mg kg^−1^ and 30 mg kg^−1^; volume: 5 mLkg^−1^). Based on previous reports on C57BL/6J mice anatomy(93), peritoneum, plasma, brain and periphery volume were set to 2 mL, 2 mL, 0.41 mL and 15 mL, respectively. Rate constants, when available, were obtained from previous pharmacokinetic studies of escitalopram(94, 95).

Simulated brain escitalopram concentration was used to generate synthetic FSCV signals of serotonin release and reuptake assuming escitalopram only affects serotonin reuptake *via* pure competitive inhibition of SERTs. First, Equation 2 was fitted to the control signals. The concentration of escitalopram ([Escit.]) was then simulated at specific time points, and an apparent K_m_ (K^app^_m_) is calculated based on Equation 9 and assuming an inhibition constant of K_i_ = 1.1 nM for escitalopram.

(9) $$K^{app}{}_{m}=K_{m}\left(1+\frac{\left[\text{Escit}.\right]}{K_{i}}\right)$$

### Statistical Analyses

All statistical tests were performed using Python’s SciPy and Pingouin libraries. Descriptive statistics are shown as average ± SEM, and inferential significance is accepted when p < 0.050.

FSCAV time series were statistically analyzed using analysis of covariance (ANCOVA) and Tukey-Kramer multiple comparisons between slopes. Additionally, individual time points were compared using a repeated measures analysis of variance (ANOVA) and multiple comparison paired t-tests. FSCV parameters (Amp_max_ and t_1/2_) of concentration vs. time traces pre- and post-drug administration were compared using individual paired *t*-tests.

Cell culture SERT density and ASP^+^ intensity between the different time and treatment groups were compared using Kruskas-Wallis test followed by Dunn’s multiple comparisons with p-values corrected *via* Bonferroni method. All python files with statistical results are available upon request.

## Data Availability

FSCV and FSCAV analysis code, including filtering, calibration, parametric analysis (Amp_max_ and t_1/2_ estimations) and Michaelis-Menten fitting algorithms are implemented in a custom-designed open source web application, The Analysis Kid(87). The code that runs the application is freely available at https://github.com/sermeor/The-Analysis-Kid. Any additional data or information required to replicate or reanalyze the data reported in this work is available from the lead contact upon request.
